# High‐dose‐rate interstitial brachytherapy for a bulky sebaceous carcinoma of the eyelid: A case report

**DOI:** 10.1002/ccr3.2360

**Published:** 2019-08-15

**Authors:** Yoshiaki Takagawa, Naoya Murakami, Shigenobu Suzuki, Fumihiko Matsumoto, Seiichi Yoshimoto, Jun Itami

**Affiliations:** ^1^ Department of Radiation Oncology National Cancer Center Hospital Tokyo Japan; ^2^ Department of Radiology Tokyo Metropolitan Tama Medical Center Tokyo Japan; ^3^ Department of Ophthalmologic Oncology National Cancer Center Hospital Tokyo Japan; ^4^ Department of Head and Neck Surgery National Cancer Center Hospital Tokyo Japan

**Keywords:** eyelid, high‐dose‐rate, high‐dose‐rate interstitial brachytherapy, sebaceous carcinoma, toxicity

## Abstract

High‐dose‐rate interstitial brachytherapy (HDR‐ISBT) achieved excellent local control of the bulky sebaceous carcinoma of the eyelid. However, we must pay attention to dose of eyelid and cornea about late toxicity of HDR‐ISBT.

## Graphical abstract

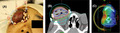

## 1 INTRODUCTION

Sebaceous carcinomas of the eyelid are uncommon and account for fewer than 1% of all eyelid tumors and approximately 5% of all eyelid malignancies in the United States.^1^, ^2^ However, sebaceous carcinomas of the eyelid are relatively common across Asia (27%‐40% of eyelid malignancies).^3^, ^4^, ^5^ Standard treatment consists of wide local excision or Mohs micrographic surgery.^6^ Some patients may be treated with radiotherapy if they are unsuitable candidates for surgery or refuse surgical intervention. Previous reports of interstitial low‐dose‐rate (LDR) and high‐dose‐rate (HDR) brachytherapy (BT) for eyelid cancer are few.^7^, ^8^, ^9^, ^10^, ^11^, ^12^, ^13^, ^14^

In the present case report, we described the presentation, treatment, and clinical outcome in a patient with a bulky sebaceous carcinoma of the eyelid treated with high‐dose‐rate interstitial brachytherapy (HDR‐ISBT). This study was approved by the institutional review board of the National Cancer Center Hospital (approval number 2017‐331) and conforms to the ethical standards laid down in the Declaration of Helsinki.

## 2 CASE REPORT

**Figure 1:**
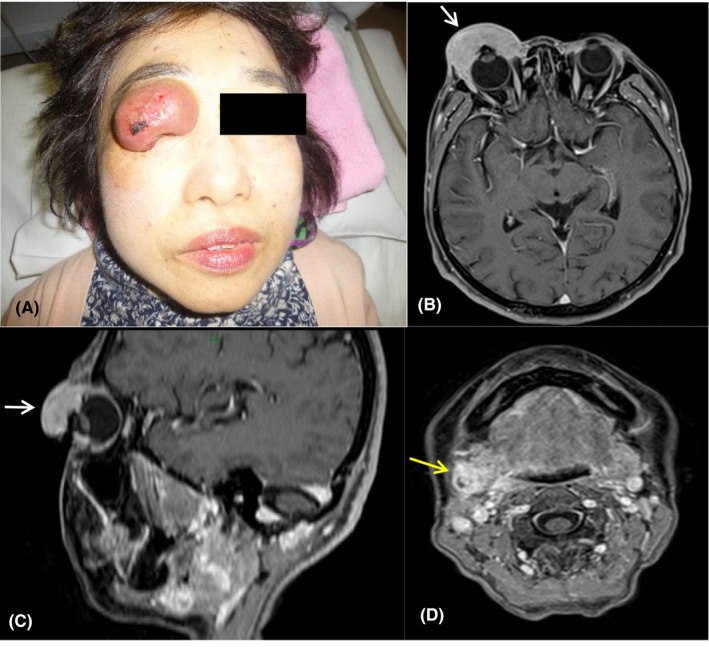
Clinical photograph of the primary tumor of the right upper eyelid (A). The right upper eyelid was replaced by a pinkish tumor with a slightly ulcerative surface showing a small amount of bleeding. Pretreatment axial MRI image (B), sagittal image (C) of the primary tumor. Right submandibular lymph node metastasis (D) (arrows)

A 61‐year‐old woman noticed a nodule in her right upper eyelid. She visited an ophthalmology clinic where an excisional biopsy was performed. The diagnosis was sebaceous carcinoma of the eyelid. The lesion was 10 mm in size. Definitive surgery was planned, but the patient refused and opted instead for an alternative treatment using medicinal herbs for 22 months. During this time, the tumor grew, and the right, submandibular lymph node became swollen. At this point, the patient realized that the herbal treatment would be unable to cure her disease and was therefore referred to our hospital. Physical examination revealed a painless, bulky, pinkish tumor in her right upper eyelid with a slightly ulcerative surface showing a small amount of bleeding (Figure 1A). We were unable to measure her visual acuity because the patient was unable to open her eye. MRI and PET findings showed that the primary tumor, measuring 4.5 × 2.9 × 2.5 cm, had infiltrated the full thickness of the eyelid. A solitary, 2‐cm, right submandibular lymph node metastasis with suspected extranodal extension was also detected (Figure 1B‐D). The stage was cT3cN1M0 (Stage IIIA) according to the UICC‐TNM 8th ed.^15^ The ophthalmologist suggested definitive surgery with exenteration and eyelid excision. However, the patient refused the surgery and was referred to the Department of Radiation Oncology. She consented to radiotherapy as the initial treatment, followed by neck lymph node dissection. We considered external beam radiation therapy (EBRT) as insufficient to control the eyelid tumor due to its size. We therefore planned HDR‐ISBT. The patient consented to this treatment after being advised of the possibility of vision loss due to HDR‐ISBT.

**Figure 2:**
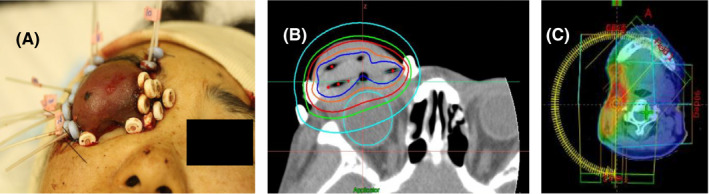
Photograph of HDR‐ISBT (A), dose distribution on CT simulator (B) (blue line: 200% isodose line; orange line: 150% isodose line; red line: 100% isodose line; green line: 80% isodose line; cyan line: 50% isodose line). Dose distribution in EBRT with IMRT for the right lymph node lesion after dissection (C)

**Figure 3:**
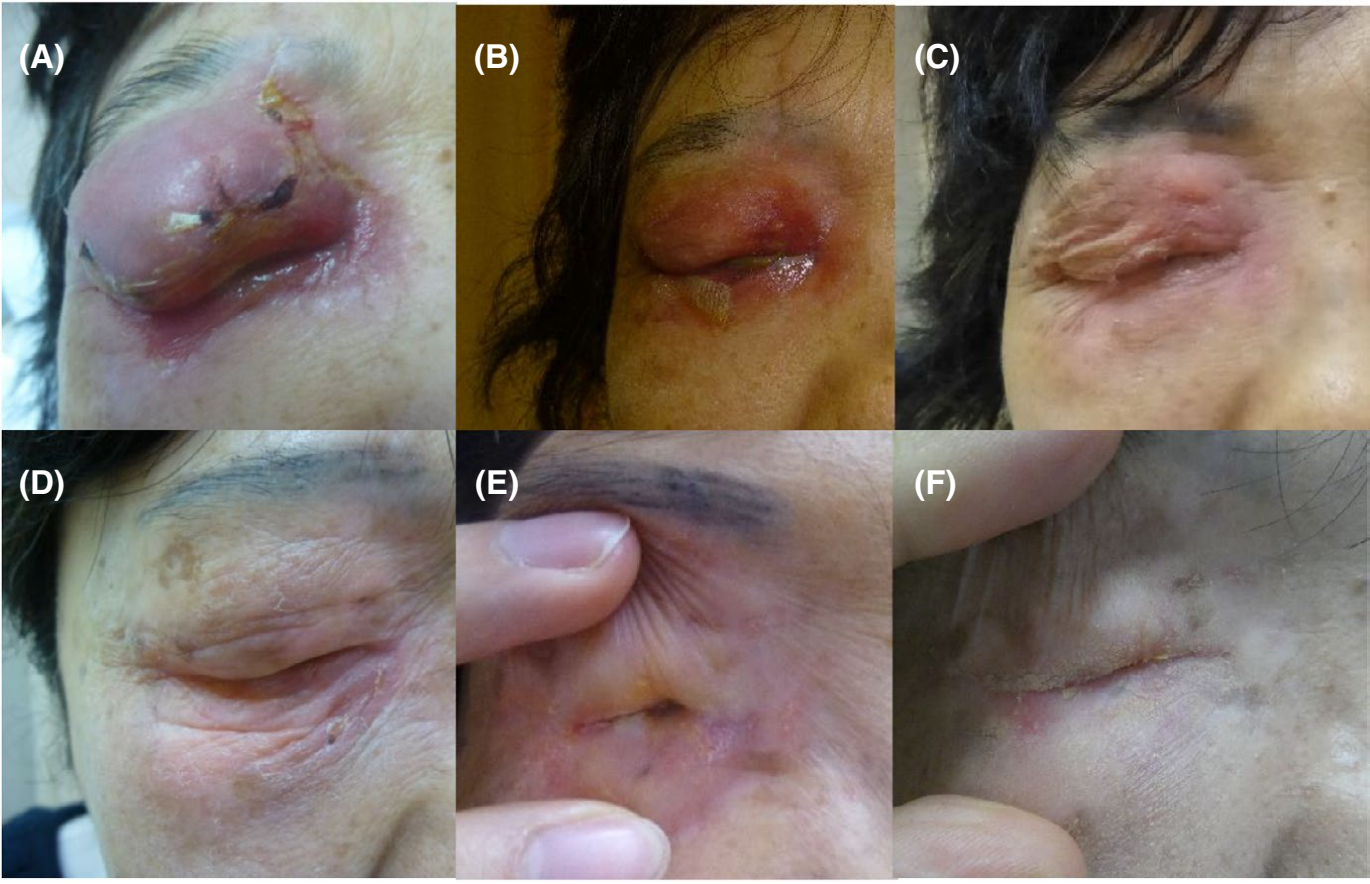
Follow‐up photographs of the primary tumor after HDR‐ISBT at (A) 1 mo, (B) 2 mo, (C) 3 mo, (D) 9 mo, (E) 12 mo, and (F) 18 mo

For remote afterloading, we used microSelectron HDR‐V3 with Oncentra Brachy (Nucletron BV) and iridium 192. Under local anesthesia, we inserted ProGuide Sharp Needle (Nucletron BV) with an outer diameter of 1.67 mm into the tumor using US and CT guidance (Figure 2A). During needle insertion, we were careful enough not to damage the eyeball. To obtain good dose distribution, we placed the interstitial needles at regular intervals in the tumor. After needle insertion, a CT simulator was used to plan treatment. To reduce the radiation dose to the cornea, we inserted a 5‐mm‐thick lead block between the eyelid tumor and right eyeball before each irradiation. Nine interstitial needles were kept in their place of insertion for five days. We irradiated the lesion twice daily at intervals of at least six hours. The radiation dose was 54 Gy in nine fractions. The dose distribution is shown in Figure 2B. The median dose of 0.1 cm^3^ in the cornea (D0.1cm^3^) was 7.8 Gy per fraction as calculated under CT simulation without a lead blocker. One month after the HDR‐ISBT, right neck lymph node dissection and partial excision of the right parotid gland were performed in the Department of Head and Neck Surgery. Pathological findings showed a solitary lymph node metastasis with extranodal extension as expected. Adjuvant prophylactic EBRT for the right lymph node lesion (right level Ib, II, and III) 16 with intensity‐modulated radiotherapy (IMRT) was performed (Figure 2C). The radiation dose was 66 Gy in 33 fractions. The primary tumor responded very well and decreased in size. The follow‐up images are shown in Figure 3. Grade 2 dermatitis was the only acute, radiation‐related toxicity.

**Figure 4:**
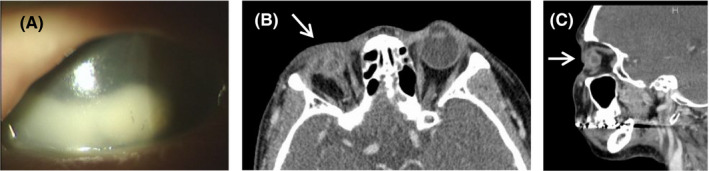
Photograph of the right corneal ulcer at 4 mo after HDR‐ISBT (A). Posttreatment axial (B) and sagittal (C) CT images of the primary tumor. Follow‐up CT scan shows phthisis of the right eyeball (arrows)

Four months after HDR‐ISBT, a follow‐up MRI detected a solitary tumor in the right residual parotid gland. This relapse of the lymph node metastasis occurred outside the external beam radiation field. At this time, the patient complained of slowly worsening pain in her right eye. She had erosion of the right cornea and received the diagnosis of a corneal ulcer from the ophthalmologist (Figure 4A). However, the eye pain improved through conservative treatment with steroid instillation. A salvage lymphadenectomy of the right parotid gland was also performed under local anesthesia by a head‐and‐neck surgeon. At this time, there was no extranodal extension in the pathological findings, and adjuvant therapy was therefore not administered.

At 18 months after HDR‐ISBT, there was no locoregional recurrence or eye pain on physical examination or CT (Figure 4B‐C). However, symblepharon with ankyloblepharon of the right eyelid appeared one year after HDR‐ISBT and gradually worsened, progressing to complete ankyloblepharon of the right eyelid (Figure 3). Follow‐up CT showed phthisis bulbi of the right eye (Figure 4B‐C).

## 3 DISCUSSION

We were able to achieve excellent local control of the primary tumor in this case by HDR‐ISBT. Additionally, combining surgical treatment and EBRT enabled good control of the neck lymph node metastasis.

Sebaceous carcinomas are associated with a disease‐related mortality rate of 6% to 30%.^17^, ^18^ These tumors can directly invade adjacent organs such as the eyeball and brain. Lymph node metastasis and distant metastasis can occur, and cases of local recurrence have been documented. Standard treatment consists of wide local excision or Mohs micrographic surgery, which enables local control in 60%‐90% of cases.^6^, ^19^, ^20^, ^21^ Erovic et al analyzed 33 patients with periorbital sebaceous carcinomas treated with primary surgery and reported that the 5‐year local and regional control rates were 63% and 58%, respectively.^21^ However, due to advanced age, comorbidities or refusal to undergo surgical intervention, some patients are unsuitable candidates for surgery. Furthermore, despite recent progress in reconstructive surgery, eyelid tumors may be difficult to excise completely without functional and cosmetic impairment.^22^, ^23^

Radiotherapy is an alternative treatment option for patients who refuse or are otherwise unsuitable for surgery. In general, most radiotherapy for sebaceous carcinomas of the eyelid consists of EBRT due to its lower burden. However, occasionally, we experience cases of the local recurrence of bulky tumors treated only with EBRT. By comparison, BT has the considerable advantage of better dose distribution and less damage to normal organ tissue. Interstitial LDR BT for eyelid carcinomas was reported by several authors 7, 8, 9 and is known to be a good option as definitive treatment for postoperative positive surgical margins or as adjuvant therapy for the early stages of eyelid cancer. However, there are few previous reports of HDR‐ISBT for eyelid carcinomas, and the sample sizes in these reports are small.^10^, ^11^, ^12^, ^13^, ^14^ Azad et al reported eight patients with sebaceous carcinoma of the eyelid treated with radical HDR‐ISBT (39 Gy/6 fr) and a 5‐year local control rate of 57.4%.^11^ Laskar et al reported eight patients with eyelid cancer (four patients with a sebaceous carcinoma) treated with postoperative adjuvant HDR‐ISBT (3‐3.5 Gy per fraction, 7‐10 fractions) and a local control rate of 100%; however, two of the patients experienced a nodal relapse.^13^ In the present case, we considered EBRT alone as insufficient to control the bulky eyelid tumor and therefore planned HDR‐ISBT.

Sebaceous carcinomas of the eyelid often relapse in the lymph nodes of the neck, particularly the preauricular, parotid, and submandibular lymph nodes, in 8% to 32% of patients.^6^, ^19^ In the current case, the patient had a solitary, right submandibular lymph node metastasis at presentation. After the initial treatment, she also had intraglandular lymph node recurrence in the right, residual parotid. By combining surgical treatment and EBRT, we were able to achieve good, locoregional control. Nonetheless, whenever possible, treatment for an N1 sebaceous carcinoma of the eyelid should be surgery for the primary tumor and neck lymph node dissection followed by adjuvant EBRT as per the pathological findings.

The present patient experienced Gr2 dermatitis as an acute toxicity and complete ankyloblepharon and phthisis bulbi as late toxicities. Mareco et al reported an occurrence rate of 76% for Gr1‐3 radiodermatitis and 42% for Gr1 conjunctivitis as acute toxicities following HDR‐ISBT treatment in 17 patients with eyelid cancer.^14^ They also stated that madarosis was the most common late toxicity and that cosmetic outcomes were good to excellent in 70% of their patients. Unfortunately, in the present case, the cosmetic outcome was poor due to phthisis bulbi of the right eye. To reduce the corneal dose, we inserted a lead blocker between the eyelid tumor and right eyeball before each irradiation. The median D0.1 cm^3^ in the cornea was 7.8 Gy per fraction calculated under CT simulation without a lead blocker. Therefore, we considered the true corneal dose with the lead blocker to be less than 7.8 Gy per fraction (the lead blocker prevented calculation of the dose by CT simulation). However, we were unable to insert a sufficiently thick and wide lead blocker due to the narrowness of the space. As a result, the exposed areas around the lead blocker received an increased dose of radiation and were damaged. If EBRT had been used after HDR‐ISBT as a boost, the late toxicity might have been milder, and her right vision might have been spared irrespective of tumor control. Late toxicities resulting from the radiation dose to the eyeball, cornea, and eyelid itself are potential hazards in HDR‐ISBT for the treatment of bulky eyelid tumors. Therefore, although we were able to achieve excellent local control, our approach was only one of the available options and possibly not the optimal choice in this case. The limitation of this case report is that there is no correct choice in the method or sequence of radiation therapy. Choosing the best treatment option from among EBRT alone, EBRT + HDR‐ISBT, and HDR‐ISBT for a bulky eyelid carcinoma is challenging. In most cases, the choice of treatment depends on comprehensive clinical assessment of the tumor size, rate of growth, patient's age, PS, etc

## 4 CONCLUSION

In conclusion, we were able to achieve excellent, local control of a bulky sebaceous carcinoma of the eyelid using HDR‐ISBT. Combining surgery with EBRT enabled good control of the lymph node metastasis of the neck in the same patient.

## CONFLICT OF INTEREST

None.

## AUTHOR CONTRIBUTIONS

YT, NM, SS, FM, SY, JI: all participated in the diagnosis and treatment of this patient. YT: is the lead author and was additionally responsible for submission and marking appropriate edits when needed. All authors approved the final manuscript.
